# New pathogen-specific immunoPET/MR tracer for molecular imaging of a systemic bacterial infection

**DOI:** 10.18632/oncotarget.7770

**Published:** 2016-02-26

**Authors:** Stefan Wiehr, Philipp Warnke, Anna-Maria Rolle, Monika Schütz, Philipp Oberhettinger, Ursula Kohlhofer, Leticia Quintanilla-Martinez, Andreas Maurer, Christopher Thornton, Frederic Boschetti, Gerald Reischl, Ingo B. Autenrieth, Bernd J. Pichler, Stella E. Autenrieth

**Affiliations:** ^1^ Werner Siemens Imaging Center, Department of Preclinical Imaging and Radiopharmacy, Eberhard Karls University Tübingen, Tübingen, Germany; ^2^ Institute of Medical Microbiology and Hygiene, Eberhard Karls University, Tübingen, Germany; ^3^ Institute of Pathology, Eberhard Karls University Tübingen, Tübingen, Germany; ^4^ Biosciences and ISCA Diagnostics Ltd., University of Exeter, Exeter, United Kingdom; ^5^ CheMatech, Faculté des Sciences Mirande, Dijon, France; ^6^ Department of Internal Medicine II, University Hospital Tübingen, Tübingen, Germany; ^7^ Institute of Medical Microbiology, Virology and Hygiene, Rostock University Hospital, Rostock, Germany

**Keywords:** bacteria, PET/MR, in vivo imaging, 64Cu, antibody

## Abstract

The specific and rapid detection of Enterobacteriaceae, the most frequent cause of gram-negative bacterial infections in humans, remains a major challenge. We developed a non-invasive method to rapidly detect systemic *Yersinia enterocolitica* infections using immunoPET (antibody-targeted positron emission tomography) with [^64^Cu]NODAGA-labeled *Yersinia*-specific polyclonal antibodies targeting the outer membrane protein YadA. In contrast to the tracer [^18^F]FDG, [^64^Cu]NODAGA-YadA uptake co-localized in a dose dependent manner with bacterial lesions of *Yersinia*-infected mice, as detected by magnetic resonance (MR) imaging. This was accompanied by elevated uptake of [^64^Cu]NODAGA-YadA in infected tissues, in *ex vivo* biodistribution studies, whereas reduced uptake was observed following blocking with unlabeled anti-YadA antibody. We show, for the first time, a bacteria-specific, antibody-based, *in vivo* imaging method for the diagnosis of a Gram-negative enterobacterial infection as a proof of concept, which may provide new insights into pathogen-host interactions.

## INTRODUCTION

Nuclear medicine enables the early and accurate detection of inflammation and infection, with the potential for clinically diagnosing infectious diseases. Molecular imaging using positron emission tomography (PET) allows the metabolic and functional activities of living cells to be determined, and when combined with pathogen-specific tracers, permits the distinction between normal and pathological tissues [1]. A limitation of PET is the availability of pathogen-specific tracers that allow for the discrimination between different infectious etiologies and between sites of sterile inflammation [2-3]. Furthermore, most of the radiopharmaceuticals used are not pathogen-specific and accumulate at the sites of inflammation [4] thereby restricting the diagnostic capabilities of the technology.

Fluorine-18 fluorodeoxyglucose ([^18^F]FDG), the major clinical PET tracer used for the detection of malignancies, has been used for imaging of infectious diseases [5]. However, as a general indicator of metabolic activity of cells, it does not allow for the specific identification of pathogens at sites of inflammation [6]. An increasing number of innovative PET radiopharmaceuticals employing monoclonal antibodies or their fragments, peptides, and small molecules have been developed and evaluated for infectious disease imaging, mainly in preclinical settings [7], but compared to tracer development in cancer research, its use for the specific detection of pathogens is in its infancy.

The Gram-negative bacterium *Yersinia enterocolitica* (*Ye*) belongs to the family *Enterobacteriaceae* and is an important cause of gastrointestinal infections. Infections are caused by ingestion of contaminated food or drinking water and can cause severe diarrhea, enterocolitis, and mesenteric lymphadenitis [8]. In immunocompromised patients, systemic infection can lead to focal abscesses in the spleen and liver [9]. Similar clinical manifestations have been found in a murine model of systemic *Yersinia* infection, with *Ye* also being detectable in the lymph nodes, bone marrow and lungs at one day post-infection [10-11]. The virulence of *Ye* is associated with *Yersinia* adhesin A (YadA), a trimeric autotransporter that mediates cell adhesion. Its presence on the cell surface makes it an ideal biomarker candidate for the specific imaging of yersiniosis [12].

The aim of this study was to evaluate the diagnostic potential of a newly developed ^64^Cu-labeled polyclonal antibody targeting YadA for used as a PET tracer and to compare its accuracy to that of [^18^F]FDG for the detection of *Ye* infections in a well-established mouse model of yersiniosis that closely resembles the course of infection in humans [13]. In the study presented here, we demonstrate a dramatically improved sensitivity and specificity of the antibody-based tracer in detecting *Ye* infection compared to the use of [^18^F]FDG. The experimental *Ye* infection used here mimics yersiniosis in humans [13] and thus offers excellent opportunities for basic *in vivo* research on newly developed immunoPET tracers for the diagnosis of pathogen-induced inflammation.

## RESULTS

### Infection of mice with *Ye* impairs their physiological condition

**Figure 1:**
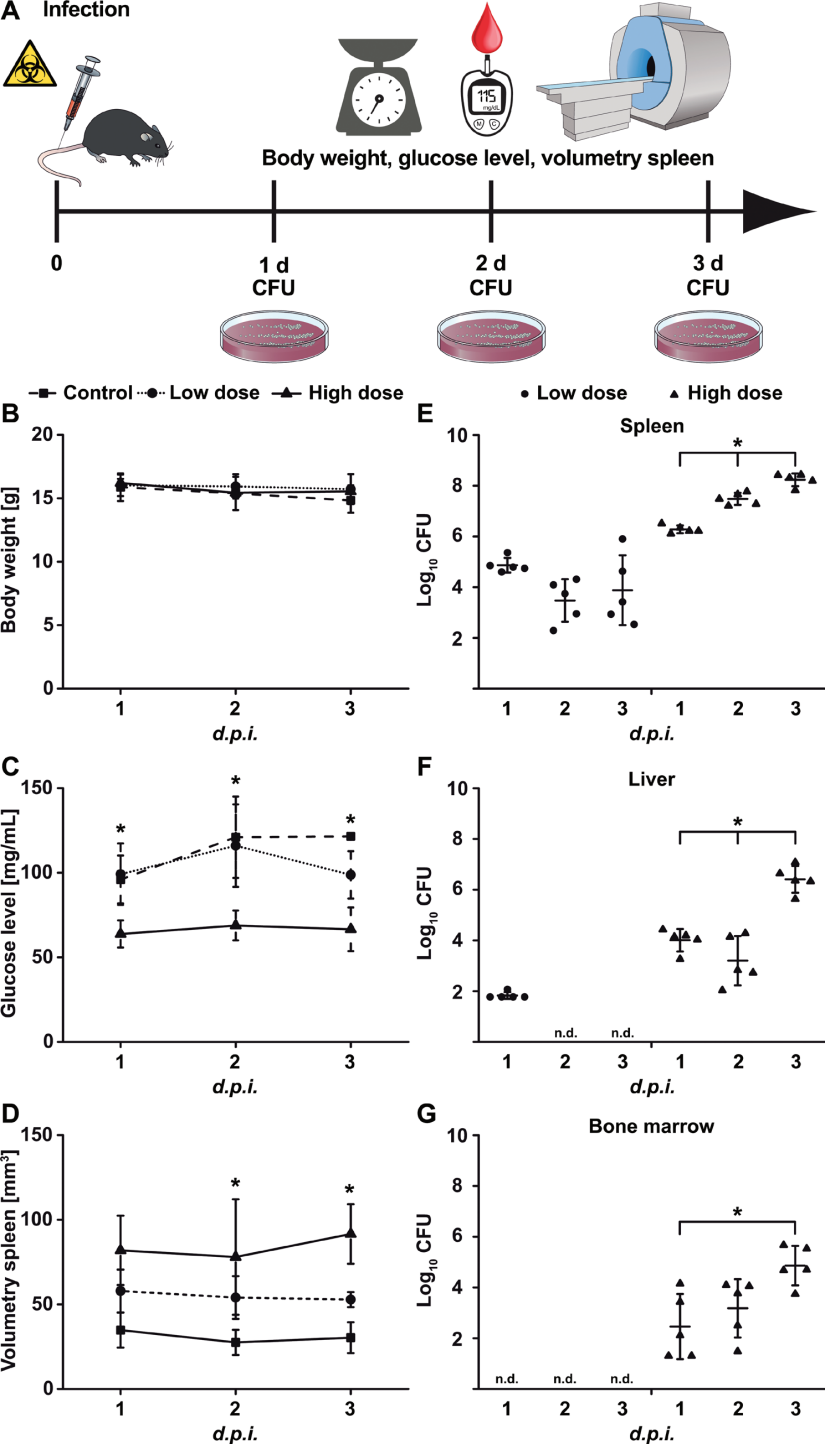
Physiological changes upon *Yersinia* infection **A.** Schematic showing the experimental procedure (scheme adapted from [46]). Mice were infected with low-dose (1×10^3^ CFU; *n* = 7) or high-dose (5×10^4^ CFU; *n* = 7) *Ye* or treated with PBS and were analyzed one to three days *p.i.* for **B.** body weight, **C.** blood glucose level, and **D.** spleen volume *via* MRI and for bacterial load (CFU) in the **E.** spleen, **F.** liver, and **G.** bone marrow. Data are shown as the means ± SD from one out of two or more independent experiments. See also, Figure S1.

To evaluate conventional PET tracers for their use in the detection of systemic *Ye* infection, we first assessed the physiological conditions of the mice after intravenous (*i.v.*) injections of two different doses of *Ye*, a sub-lethal dose (1×10^3^ *Ye,* indicated as the low dose) and a lethal dose (5×10^4^ *Ye,* indicated as the high dose), or an injection with PBS as the control (Figure 1A). No significant differences in the body weights of the different animal groups were observed throughout the infection period (Figure 1B), although marginal weight loss was observed in mice due to the infection and/or anesthesia. Throughout the infection period, the blood glucose levels and the water and food intake levels of the high-dose-infected animals were significantly lower than those of the PBS-treated and low-dose-infected mice (Figure 1C).

The spleen size of the animals was assessed *in vivo via* volumetric measurements using MRI and was found to be correlated with the severity of infection (Figure 1D). Consistent with this, the bacterial load was significantly higher in the spleens of the high-dose- than it was in the low-dose-infected mice (Figure 1E). Bacteria were also detected in the liver and bone marrow of the high-dose- but not of the low-dose-infected mice (Figure 1F and 1G). Thus, the spleen is the major focus of *Ye* replication, and moderate infection leads to the eradication of the bacteria and the subsequent recovery of the mice, while severe infection results in an overwhelming infection and death.

### PET-MR imaging of *Ye* with [^18^F]FDG

**Figure 2:**
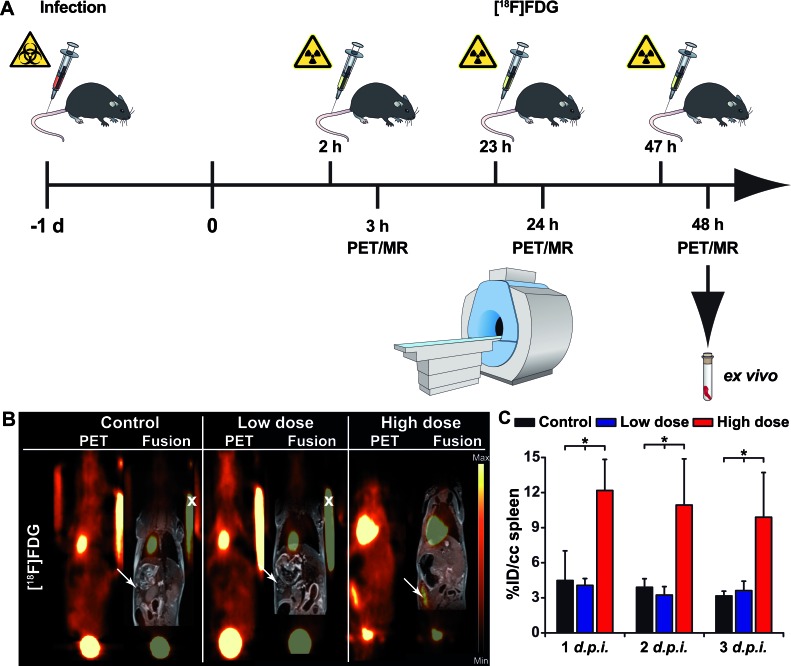
Quantification of PET images with the [^18^F]FDG tracer in *Ye*-infected mice **A.** Schematic showing the experimental procedure (scheme adapted from [46]). The mice were divided into three groups: group 1, control (not infected); group 2, low dose-infected; group 3, high dose-infected. Mice were infected with 5 × 10^4^ (high dose) or 1 × 10^3^ (low dose) CFU of *Y. enterocolitica i.v.* at day 1. The imaging protocol included sequential PET/MR imaging of uninfected control and *Ye*-infected mice on 3 consecutive days. Coronal [^18^F]FDG PET and fused PET and MR images from PBS-treated and low- and high-dose-infected mice 3 days *p.i.* **B.**. Arrows indicate the positions of the spleen. Fiducial markers used for co-registration of the PET and MR images are marked with an x. **C.** Quantitative analysis of the [^18^F]FDG uptake in the spleen is shown (black bars, PBS treated controls (*n* = 3); blue bars, low-dose-infected mice *n* = 7); red bars, high-dose-infected mice (*n* = 7-8). Data are shown as the means ± SD (%ID/cc). See also, Figure S2 and Tables S1, S2 and S3.

The clinical PET tracer [^18^F]FDG, commonly employed for oncological purposes, was used to image systemic *Ye* infection in mice. For *in vivo* evaluation of the tracer, *Ye*-infected and PBS-treated mice were *i.v.* injected with 12-14 MBq of [^18^F]FDG on days 1, 2 and 3 post-infection (*p.i.*), and PET and MRI scanning data were acquired after an uptake time of 60 min (Figure 2A). A high uptake of [^18^F]FDG in the spleen of high-dose-infected mice was observed throughout the infection. A slight increase in tracer uptake upon high-dose infection was also observed in the spine, liver and brain (Figure 2, Figure S2, Table S1). Upon infection with a low dose of *Ye*, marginal [^18^F]FDG uptake was observed in the spleen but not in other organs, and there was no difference relative to the PBS-treated mice (Figure S2 and Table S1). The PET imaging data were confirmed by an *ex vivo* biodistribution analysis after the last PET/MRI scan on day 3 *p.i.* (Table S2).

### PET-MR imaging with the *Ye*-specific tracer [^64^Cu]NODAGA-YadA

**Figure 3:**
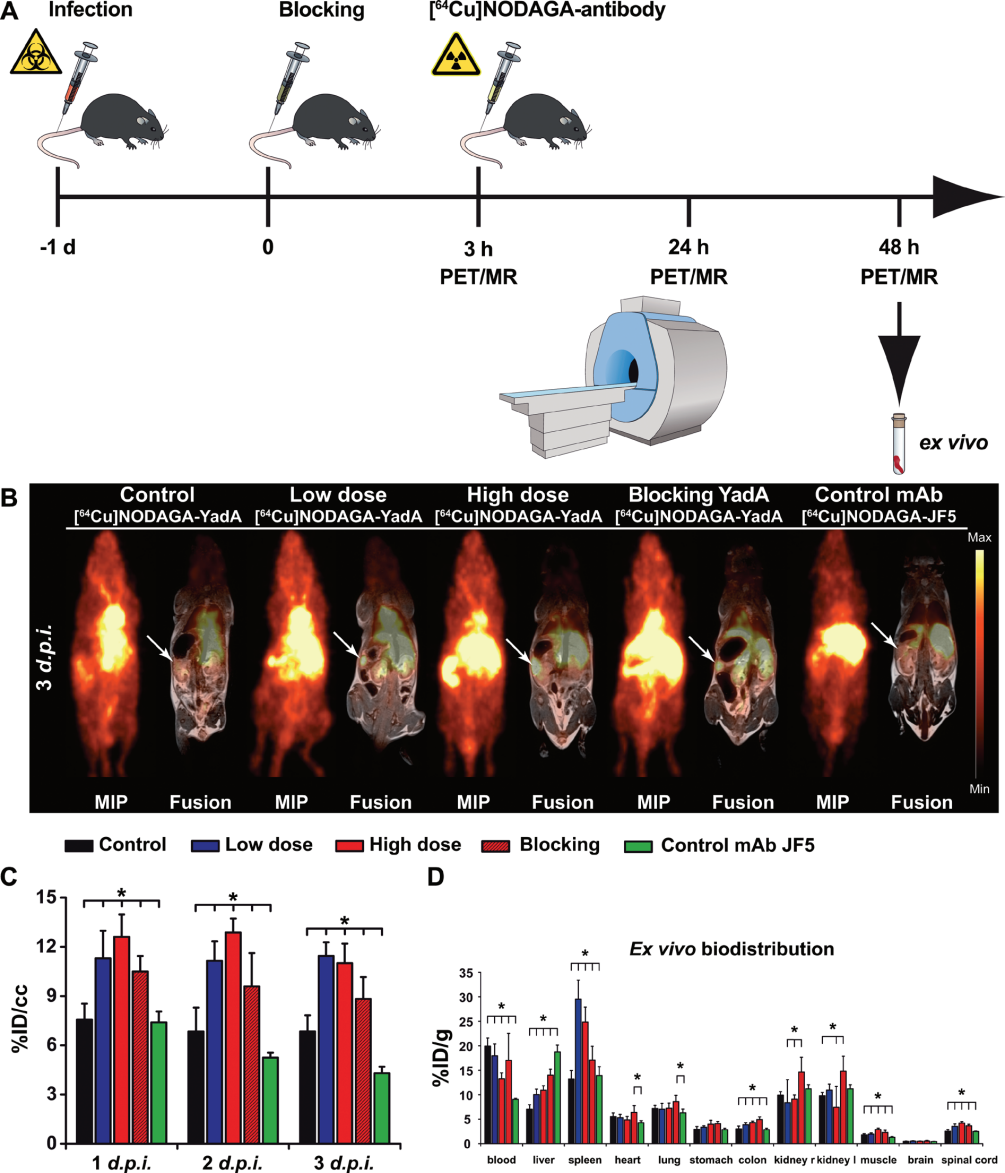
Quantification of PET images with the [^64^Cu]NODAGA-YadA tracer in *Ye*-infected mice **A.** Schematic showing the experimental procedure (scheme adapted from [46]). The mice were divided into three groups: group 1, control (not infected); group 2, low-dose-infected; group 3, high-dose-infected. Mice were infected with 5 × 10^4^ (high dose) or 1 × 10^3^ (low dose) CFU of *Y. enterocolitica i.v.* at day -1. All groups received a single *i.v.* injection with the respective [^64^Cu]NODAGA-labeled antibody and were sequentially imaged *via* PET/MR on 3 consecutive days. Coronal [^64^Cu]NODAGA-YadA PET and fused PET and MR images from PBS-treated and low- and high-dose-infected mice 3 days *p.i.* **B.**. Administration of polyclonal non-radiolabeled YadA antibody 3 h prior to the injection of [^64^Cu]NODAGA-YadA (to block YadA) or the administration of the *Aspergillus*-specific tracer [^64^Cu]NODAGA-JF5 (control mAb) into high-dose-infected mice served as the control treatments. Arrows indicate the positions of the spleens in the mice. **C.** Quantification of the PET images as measured in **B.** the quantitative analysis of [^64^Cu]NODAGA-YadA or [^64^Cu]NODAGA-JF5 uptake in the spleen is shown. **D.** *Ex vivo* biodistribution of the tracer [^64^Cu]NODAGA-YadA in the depicted organs. (black bars, PBS (*n* = 5); blue bars, low-dose *Ye* (*n* = 5); red bars, high-dose *Ye* (*n* = 5); red dashed bars, high-dose *Ye* blocked with 500 μg non-labeled YadA antibody (*n* = 5); green bars, high-dose *Ye* imaged with the *Aspergillus*-specific tracer [^64^Cu]NODAGA-JF5 (*n* = 4). Data are shown as the means ± SD. See also, Figure S3 and Tables S5 and S6.

To specifically detect *Ye* infection using PET imaging, we developed a *Ye*-specific tracer based on a ^64^Cu-labeled polyclonal antibody targeting the outer membrane protein YadA. The radiochemical purities of NODAGA-YadA and the *Aspergillus*-specific control antibody NODAGA-JF5 were 85-95 % and 65 %, respectively, after labeling with ^64^Cu. The remaining ^64^Cu was bound to unconjugated NODAGA, and the fraction of uncomplexed ^64^Cu^2+^ was below 5 % in both cases. The specific activity in two independent labeling procedures was 650-730 MBq/mg for NODAGA-YadA and 500 MBq/mg for NODAGA-JF5. For the assessment of the serum stability, one volume of [^64^Cu]NODAGA-YadA in its final formulation was incubated with three volumes of C57BL/6 serum at 37°C for various lengths of time and immediately analyzed *via* radio-HPSEC (Figure S4A) and autoradiography (Figure S4B). The radiochemical purity for up to 48 h remained above 90 %, and the analysis showed no signs of proteolytic degradation, protein aggregation or copper transchelation to serum proteins over the 48 h period under these conditions (Figure S4C). The immunoreactivity of the YadA antibody following labeling with the chelator NODAGA was investigated *via* immunofluorescence using YadA-expressing (positive) and non-YadA-expressing (negative) *Ye* or *E. coli* bacteria. The labeling of the antibody with the chelator did not alter the specific binding of the antibody to its target YadA antigen (Figure S5). Intense fluorescence of the YadA-positive *Ye* or *E. coli* and a lack of staining of the non-YadA-expressing bacteria were observed in each case. PET imaging showed a high uptake of the radiolabeled [^64^Cu]NODAGA-YadA polyclonal antibody in the spleens of low-dose- and high-dose-infected mice throughout the infection period in a dose-dependent manner. A significantly lower uptake was observed in the spleens of PBS-treated control mice (Figure 3, Figure S3). Blocking experiments were performed by injecting 500 μg of non-radiolabeled anti-YadA antibody 3 h prior to the injection of radiolabeled [^64^Cu]NODAGA-YadA into high-dose-infected mice to determine the *in vivo* specificity of the newly developed PET tracer. Blocking was already evident 24 h after the injection, with significantly lower uptake of the radiolabeled tracer observed in the spleens of mice treated with the blocking antibody than were observed in the spleens of the non-blocked high-dose-infected mice (Figure 3B, Figure S3; Table S3). The tracer [^64^Cu]NODAGA-JF5, which included the *Aspergillus*-specific monoclonal antibody JF5 [14], was used as an additional control and showed significantly lower uptake than was observed in the other treatment groups (Figure 3). The quantified results for the tested organs are shown in Supplement Table S3 and were confirmed by the *ex vivo* biodistribution data (Figure 3C and Table S4).

### Immunohistochemistry and antibody-based analysis

**Figure 4:**
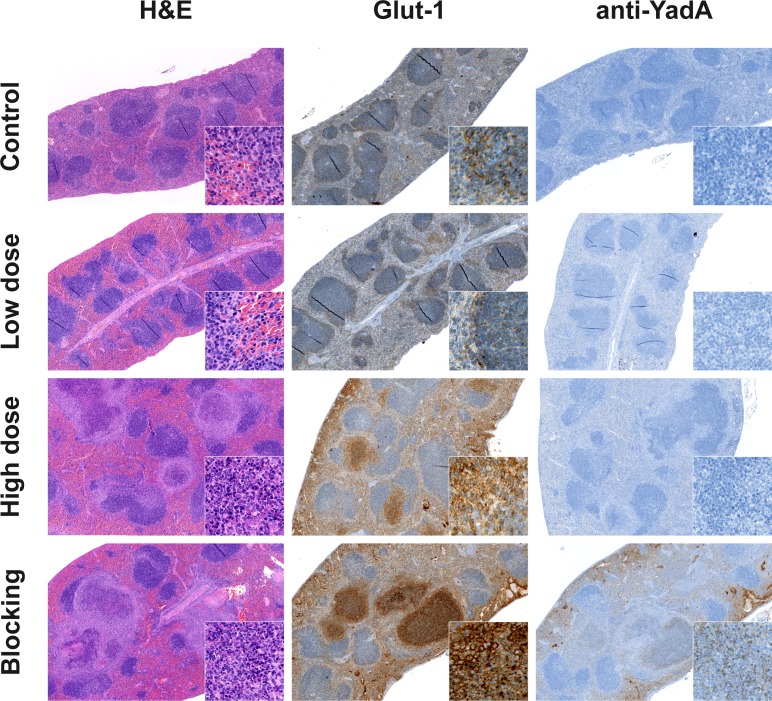
Immunohistochemistry supports the data from PET imaging Immunohistochemical staining of spleen tissue sections from PBS-treated, low-dose- and high-dose-infected mice 3 days *p.i.* Administration of the polyclonal non-radiolabeled antibody YadA 3 h prior to injection of the [^64^Cu]NODAGA-YadA (to block YadA) into high-dose-infected mice served as control treatment. Sections were stained for H&E, Glut-1, and the goat anti-rabbit secondary antibody to detect the *in vivo* administered YadA (blocking) antibody. All the magnifications are indicated. Inserts show Glut-1 (630x) and the anti-YadA Ab (400x). Data are representative of 12 analyzed mice.

To confirm the PET results, sections of the spleens from all groups of mice were analyzed *via* immunohistochemistry. Macroscopically, the PBS-treated and low-dose *Ye*-infected mice had normal sized spleens, whereas the spleens of the high-dose-infected animals were enlarged. Additionally, the spleens of the high-dose-infected mice showed signs of lymphoid hyperplasia, with germinal centers and multiple abscesses detected by H&E staining (Figure 4).

Spleens were further analyzed for Glut-1 expression, an indicator of the degree of glucose metabolism. Slight Glut-1 staining was observed in the periphery of the splenic white pulp of the PBS-treated and low-dose-infected animals, with no difference detectable between these groups. In contrast, Glut-1 expression was elevated, especially in the necrotic areas of abscesses, in the spleens of high-dose-infected animals (Figure 4). These data support the results obtained from the [^18^F]FDG imaging that showed increased FDG uptake in the spleens of high-dose-infected mice but not in those from low-dose-infected mice.

To confirm the blocking experiment with the YadA antibody applied prior to the *Ye*-specific tracer, splenic sections were stained with a secondary antibody that detects the blocking antibody. Positive staining for this antibody was only observed in the abscesses caused by *Ye* in the sections of spleens from mice treated with the blocking antibody YadA ([500 μg/mouse]). The concentration of the *Ye* tracer [^64^Cu]NODAGA-YadA was too low ([20 μg/mouse]) to be detected in the spleens of the low-dose and high-dose infected animals *via* immunohistochemistry.

## DISCUSSION

Infectious pathogens are a serious health issue, and their accurate detection remains a major challenge in medicine. Effective treatment relies on pathogen identification at an early stage of the infection, and yet, many infections remain undiagnosed prior to their systemic manifestation [7]. Nuclear imaging has emerged as a rapid, non-invasive and highly sensitive approach to disease diagnosis, which can identify sites of infection and inflammation more rapidly than conventional laboratory-based diagnostic techniques [15]. These traditional diagnostic modalities often result in long turn-around times, making it more difficult when dealing with contaminants, thereby, compelling clinicians to treat patients empirically with broad-spectrum antibiotics until diagnostic results are available [16-17].

Existing tracers for PET imaging are not able to distinguish between malignancies and sterile or pathogen-induced inflammations [18-19]. Furthermore, at the late stages of an infection, it is even more challenging to correctly diagnose the cause of the illness because the disease can manifest non-pathogen-induced symptoms similar to those observed in malignancies [20]. Here, we present a novel pathogen-specific imaging tracer for use in the non-invasive dose-dependent detection and diagnosis of a bacterial infection of humans. Enterobacteriaceae are the most common cause of Gram-negative bacterial infections. To investigate the specificity of our tracer, we used a well-established [21] Enterobacteriaceae-sepsis mouse model, with the intravenous administration of *Ye*. Consistent with previous studies, the pathogen was mainly found within the spleen, liver and, to a lesser extent, bone marrow [10]. The radiolabeled anti-YadA antibody, which binds to the outer membrane protein *Ye* adhesin A (YadA), was able to specifically detect the pathogen *in vivo*. Notably, the anti-YadA antibody used in this study targets a pathogenic factor essential for *in vivo* virulence, highlighting the specificity of the newly developed PET tracer. [^64^Cu]NODAGA-YadA uptake occurred in an infection dose-dependent manner, demonstrating the sensitivity of the new tracer and its ability to discriminate lethal and sub-lethal infections. Furthermore, a constantly elevated uptake of the [^64^Cu]NODAGA-YadA tracer was observed over time in low-dose- and high-dose-infected animals, independent of their bacterial burden. In contrast, elevated uptake of the standard PET tracer [^18^F]FDG was only observed in the spleen of high-dose *Ye*-infected mice, which was accompanied by a slight increase in [^18^F]FDG uptake by the spine, liver and brain. However, blood glucose levels were elevated in both low- and high-dose-infected animals. These results indicate that while the tracer [^18^F]FDG is able to detect severe inflammation in high-dose-infected mice, glucose metabolism during sub-lethal infections appears to not be increased as shown by consistently unaltered tracer uptake. This may be explained by the induction of inflammation and the increased [^18^F]FDG uptake caused by activated immune cells at the infection site [6, 22] rather than by the bacteria themselves, as there was no correlation with the bacterial loads. The comparable ^18^[F]FDG uptake in the spleen of low-dose-infected and uninfected mice is probably due to the physiological uptake pattern of [^18^F]FDG in this organ resulting in a ‘false negative’ detection of the tracer in low-dose-infected mice. This is in agreement with the findings of other groups that have shown that [^18^F]FDG is nonspecific in PET oncological applications [23-24]. Furthermore, [^18^F]FDG is unable to detect early stage bacterial infections and, in some instances, shows increased signal intensities after effective antibiotic treatments [25]. Nevertheless, it is currently the principal tracer used for the imaging of infectious diseases in humans [20, 26].

The application of whole-body immunoPET imaging to the field of infectious diseases is in its infancy [27]. It has been most recently used to detect SIV (simian immunodeficiency virus) infections in macaques [28]. For bacterial infections, several PET tracers using antibiotics, peptides, antibodies and radiolabeled white blood cells have been developed and tested in preclinical studies, but so far, they have had a limited clinical impact [18, 29]. More recently, sugar transporters for sorbitol and maltose or an alternative sugar transporter, the bacterial universal phosphate transporter (UHPT), have been investigated as targets for bacteria-specific imaging using 2-[^18^F]fluorodeoxysorbitol ([^18^F]FDS) [17], 6-[^18^F]fluoromaltose (MH^18^F) [29], and an analogue of glucose, [^18^F]FDG-6-P [30]. Using these tracers, it is possible to distinguish between Gram-negative (e.g., Enterobacteriaceae) and Gram-positive (e.g., *Staphylococcus aureus*) bacteria. [^18^F]FDS has recently been shown to be highly sensitive in the detection of *E. coli,* with a detection limit of 6.2 ± 0.2 log_10_ CFU [17]. In contrast, [^64^Cu]NODAGA-YadA allowed the detection of as few as 3.5 ± 0.4 log_10_ CFU *Ye* in the spleen, demonstrating the high sensitivity of our tracer. Recently, an *Aspergillus*-specific monoclonal antibody has been used to enable the use of immunoPET/MR imaging for the diagnosis of invasive pulmonary aspergillosis (IPA). The PET tracer [^64^Cu]DOTA-JF5 was able to distinguished IPA from bacterial lung infections and, in contrast to [^18^F]FDG-PET, was able to discriminate IPA from a general increase in metabolic activity associated with lung inflammation. This work demonstrates the applicability of molecular imaging for antibody-guided detection and its potential for aiding clinical diagnoses and the management of infectious diseases [31]. The highly *Aspergillus*-specific NODAGA-labeled monoclonal antibody JF5 was used in this study as a *Ye* nonspecific control antibody. In general, intact antibodies are highly specific molecules and constitute, along with other molecules, the first-line of defense against pathogens. The bivalent binding properties of antibodies provide a high affinity and avidity for their specific antigens. Antibodies for immunoPET, labeled directly or *via* chelators, possess great potential for disease-specific imaging and are already used in clinics for immunoPET imaging, mainly for cancer diagnostics [32-34]. The ideal imaging agents should be characterized by an intrinsic serum stability, good target uptake and persistence, non-immunogenicity, and optimal clearance from the circulation to obtain the highest signal differences [33, 35]. Native antibodies (150 kDa) are known to have a long serum half-life, possibly causing inadvertent irradiation and toxicity to healthy tissue when radiolabeled and used as immunoPET tracers [36]. One approach to overcome these drawbacks is to reduce the size of the antibodies. Using genetic engineering, defined antibody fragments can be constructed and produced [34, 37]. The reduced size of mAb fragments leads to a much faster serum clearance, which results in higher target-to-reference-tissue ratios at earlier time points compared to results obtained from the use of parental antibodies. The disadvantages of radiolabeled antibody fragments are their accumulation in the kidneys, which can possibly cause high radiation doses [38-39] similar to standard small molecule tracers used in clinics, and their varying degree of specificity for the detection of infections. An advantage of the long *in vivo* serum half-life of full length antibodies is that, when used as immunoPET tracers, they allow for the repeated imaging of patients following a single injection of the radiolabeled tracer, thereby, enabling the monitoring of disease progression, pathogen dissemination and responses to treatment.

A limitation of the present study was that a clear identification of the pathogen in the liver tissue was not possible due to the nonspecific uptake of the radiolabeled antibody and the free-copper accumulation, which resulted in a high PET signal. The nonspecific uptake of radiolabeled antibodies in the liver is a common finding [40-42]. Thus, in this organ, nonspecific uptake cannot be distinguished from specific [^64^Cu]NODAGA-YadA uptake by *Ye*.

Our work is, to the best of our knowledge, the first example of immunoPET imaging for the rapid, sensitive and specific detection of infection by a human enteropathogenic bacterium. Thus, we show a proof of concept for the use of [^64^Cu]NODAGA-labeled pathogen-specific antibodies as candidates for use as imaging probes in preclinical testing. We propose that our approach using [^64^Cu]NODAGA-anti-YadA could be adapted to detect other bacterial pathogens *via* immunoPET. Future efforts should also focus on class-specific antibodies for the detection and differentiation of pathogenic bacteria.

## MATERIALS AND METHODS

### Mice, infection and bacterial load

All animal procedures were carried out according to protocols approved by the Regierungspräsidium Tübingen (IZ1/10). Female C57BL/6JOlaHsd mice were infected with 5 × 10^4^ (high dose) or 1 × 10^3^ (low dose) CFU of *Ye* WA-314 (serotype 0:8) *via* injection into the tail vein. Mice were sacrificed 1, 2 or 3 days *p.i.* and the organs were weighed. Some portions were frozen for histology and other portions were homogenized to determine the bacterial load, which was obtained after plating serial dilutions of the cell suspensions on Müller-Hinton agar plates. For PET/MR imaging, a separate group of mice was used and analyzed on 3 consecutive days.

### PET tracer production

Fluorine-18 was produced as [^18^F]fluoride in a PETtrace cyclotron using the ^18^O(p, n)^18^F nuclear reaction, and [^18^F]FDG was synthesized as described elsewhere [43]. To generate the *Ye*-specific PET tracer, whole serum from rabbits that had been immunized with purified YadA protein [44] was used for the purification of the IgG fraction. NODAGA NHS Ester (Chematech, Dijon, France) was conjugated with the antibody in Chelex 100 (Sigma-Aldrich) treated phosphate-buffered saline at a molar ratio of 55:1 for 18 h. Excess chelator was removed using seven sequential ultrafiltration steps with PBS. For radiolabeling, copper-64 was produced as described previously [45] and incubated with the NODAGA-conjugated antibody for 1 h at 42°C.

### Serum stability of the chelator-conjugated YadA antibody

For serum stability tests, one volume of [^64^Cu]NODAGA-YadA (after clean-up with a Bio-Spin 6 column) was incubated with three volumes of C57BL/6 serum at 37°C. Samples were removed after 0 h, 1 h, 3 h, 24 h and 48 h and immediately analyzed using radio-HPSEC. Retention times for void volume, reference IgG and internal volume were 3.34 min, 5.67 min and 8.82 min, respectively. In addition, samples were run on iTLC-SG paper with 0.1 M sodium citrate (pH 5) and analyzed *via* autoradiography.

### PET/MR imaging and *ex vivo* biodistribution

The imaging protocol included sequential PET/MR imaging of uninfected control and *Ye*-infected mice on three consecutive days. The animals were treated/infected as described above and scanned on days 1, 2 and 3 *p.i.* Mice were imaged using a small-animal PET scanner (Inveon, Siemens Preclinical Solutions, Knoxville, TN, USA), which yielded a spatial resolution of approximately 1.3 mm in the reconstructed images. All of the animals were briefly anaesthetized with 1.5 % isoflurane mixed with 100 % oxygen at a flow rate of 0.8 L/min and injected *i.v.* with 12-14 MBq of [^18^F]FDG *via* a lateral tail vein. For [^18^F]FDG imaging, mice were kept under anesthesia with 1.5 % isoflurane mixed with 100 % oxygen at a flow rate of 0.8 L/min in a 37°C heated anesthesia chamber for 60 min. For immunoPET imaging, mice were injected with 20 μg of either the [^64^Cu]NODAGA-labeled anti-YadA antibody or the [^64^Cu]NODAGA-labeled *Aspergillus*-specific JF5 control mAb, which corresponded to 12-14 MBq. For the blocking studies, 500 μg of the non-radiolabeled YadA antibody was injected 3 h prior to the injection of the tracer. Ten-minute static PET scans were acquired after the uptake time of the F-18 labeled tracer (Figure 2A) and 3, 24 and 48 h after the injection of the [^64^Cu]NODAGA-labeled tracers (Figure 3A). During the PET and magnetic resonance (MR) imaging, the animals were anesthetized with 1.5 % isoflurane mixed with 100 % oxygen. PET data were acquired in list-mode, histogrammed in one 10-min time frame and reconstructed using an iterative ordered subset expectation maximization (OSEM) algorithm. No attenuation corrections were applied. MR imaging was performed on a 7 T small animal MR tomography system (Clinscan, Bruker Biospin MRI, Ettlingen, Germany) to obtain anatomical information. A T2-weighted 3D-space sequence (TE / TR 202 / 2500 ms, image matrix of 137 × 320, slice thickness 0.27 mm) was used for whole-body imaging. PET images were normalized to each other and subsequently fused to their respective MR images and analyzed using the Inveon Research Workplace software (Siemens Preclinical Solutions). Regions of interest (ROIs) were drawn around the respective tissues based on the anatomical information from the MR images. Volumetric data from the spleens were assessed based on the PET/MR data using these ROIs. Absolute quantification of the PET data are expressed as a percentage of the injected dose (%ID/cc). Blood was collected *via* a retrobulbar puncture from the anesthetized mice prior to tracer administration, and glucose was immediately measured in a HemoCue glucose system (HemoCue GmbH, Grossostheim, Germany).

After the final PET scan, all animals were sacrificed by cervical dislocation under deep anesthesia and dissected. Organs were removed and levels of radioactivity were quantified using an aliquot of the injected radiotracer in a γ-counter (Wallac 1480 WIZARD 3” Gamma Counter; Perkin Elmer, Waltham, MA, USA) using an energy window between 350 and 650 keV. The results are expressed as % of the injected dose per g (%ID/g) of tissue or spleen-to-muscle ratios.

### Histology

All organs were fixed in 4 % formalin and embedded in paraffin. For histological analyses, 3-5 μm-thick sections were prepared and stained with hematoxylin and eosin (H&E). Immunohistochemistry was performed on an automated immunostainer (Ventana Medical Systems, Inc.), according to the manufacturer's protocols for open procedures with slight modifications. All slides were stained with antibodies against Glut-1 (Abcam Inc., Suite B2304 Cambridge, USA). To detect the primary *Y. enterocolitica* YadA antibody a biotinylated anti-rabbit IgG secondary antibody (Vector Laboratories, Inc., Burlingame, USA) was used. Appropriate positive and negative controls were included to confirm the accuracy of the staining.

### Immunofluorescence microscopy

For immunofluorescence staining, 2 × 10^7^ bacteria were centrifuged on polyethyleneimine-coated coverslips, fixed for 60 min with 4 % PFA in PBS (w/v) and subsequently blocked overnight with 1 % bovine serum albumin (BSA) in PBS (w/v) at room temperature. Staining was performed using polyclonal rabbit antibodies directed against YadA or [^64^Cu]NODAGA-YadA (1 mg/ml each, diluted 1:5 in PBS), which were incubated with samples for 2 h in a dark chamber with a humidified atmosphere at room temperature. A 1:100 dilution of a Cy2-conjugated secondary goat-anti-rabbit IgG antibody (Dianova, Hamburg, Germany) was applied for 2 h at room temperature. Finally, cover slips were mounted with Mowiol. Fluorescent images were obtained using an upright DMRE fluorescence microscope (Leica, Wetzlar, Germany) equipped with a Leica b/w digital camera using the 100x objective, optovar 1.6x and the Leica application suite software. All samples from the experiment were recorded using identical software settings (exposure, gamma correction). Images were processed and assembled into figures using Adobe Photoshop/Illustrator.

### Statistical analysis

Statistical significance was determined using a two-tailed *t*-test. For experiments investigating more than two groups, statistical significance was calculated using a one-way analysis of variance (ANOVA) followed by Tukey's multiple comparison test conducted with Origin 8 software (OriginLab Corporation, Northampton, MA, USA). Data were considered statistically significant at *p* < 0.05. All quantitative data are shown as the mean ± 1 standard deviation (SD). For *t*-tests, group sizes were determined based on reaching statistical significance at a 5 % threshold and a power of 90 %, with the average values differing by 2 SD. In all multi-group comparisons, the statistical significance threshold of 5 % was adjusted based on Tukey-Kramer corrections according to group numbers.

## SUPPLEMENTARY MATERIAL FIGURES AND TABLES


