# Supplementary material for: New pathogen-specific immunoPET/MR tracer for molecular imaging of a systemic bacterial infection
Source: Oncotarget. 2016 Feb 26;7(10):10990–1001. doi: 10.18632/oncotarget.7770 (PMC4905453; doi:10.18632/oncotarget.7770)
Supplement: Supplementary file 1 [file oncotarget-07-10990-s001.pdf]

New pathogen-specific immunoPET/MR tracer for molecular imaging of a systemic bacterial infection

Supplementary Material

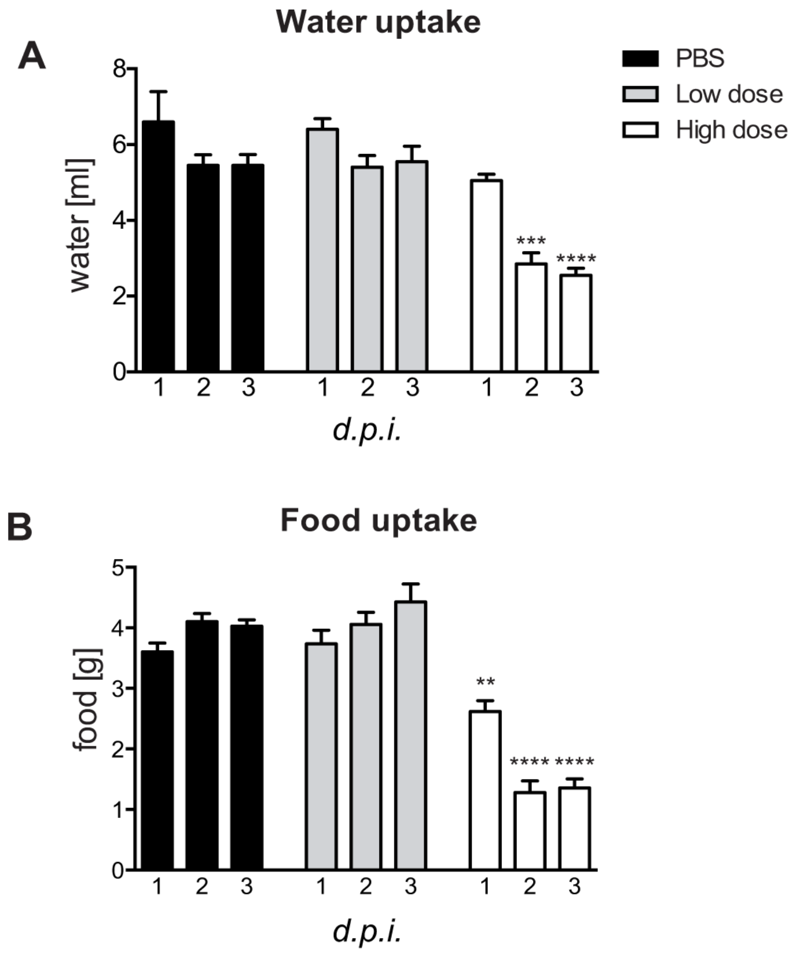

Supplemental Figure 1: Mice were infected with $1x10^3$ CFU (low dose) or $5x10^4$ CFU (high dose *Y. enterocolitica* or treated with PBS and analyzed one to three days post infection for uptake of (a) drinking water and (b) food uptake per mouse per day (n = 5; mean +/- SEM; ANOVA /Sidak's multiple comparison test).

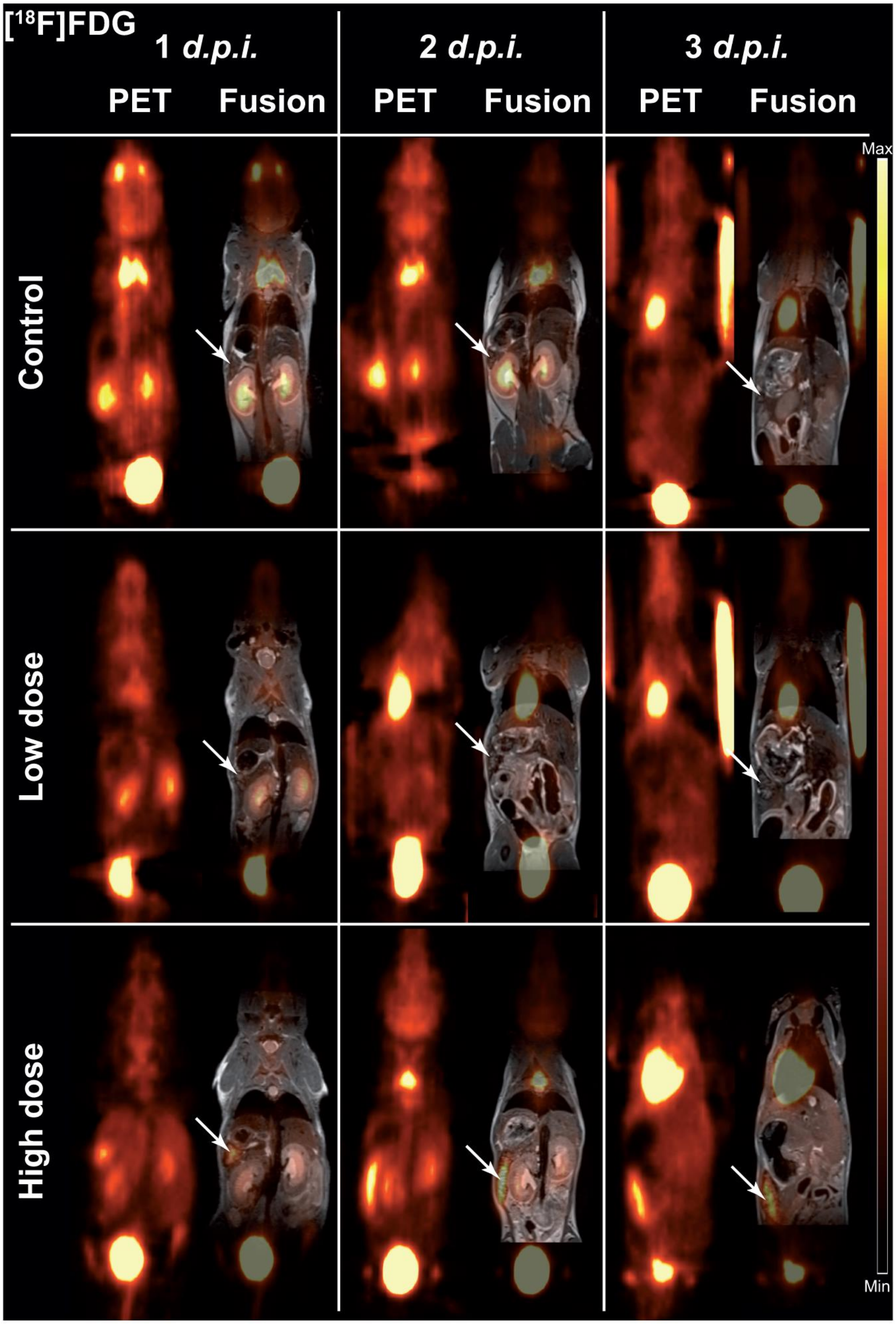

Supplemental Figure 2: Coronal [^{18}F]FDG PET and fused PET and MR images (Fusion) from PBS treated control, low and high dose infected mice 1, 2 or 3 *d.p.i*. Arrows indicate the position of the spleen.

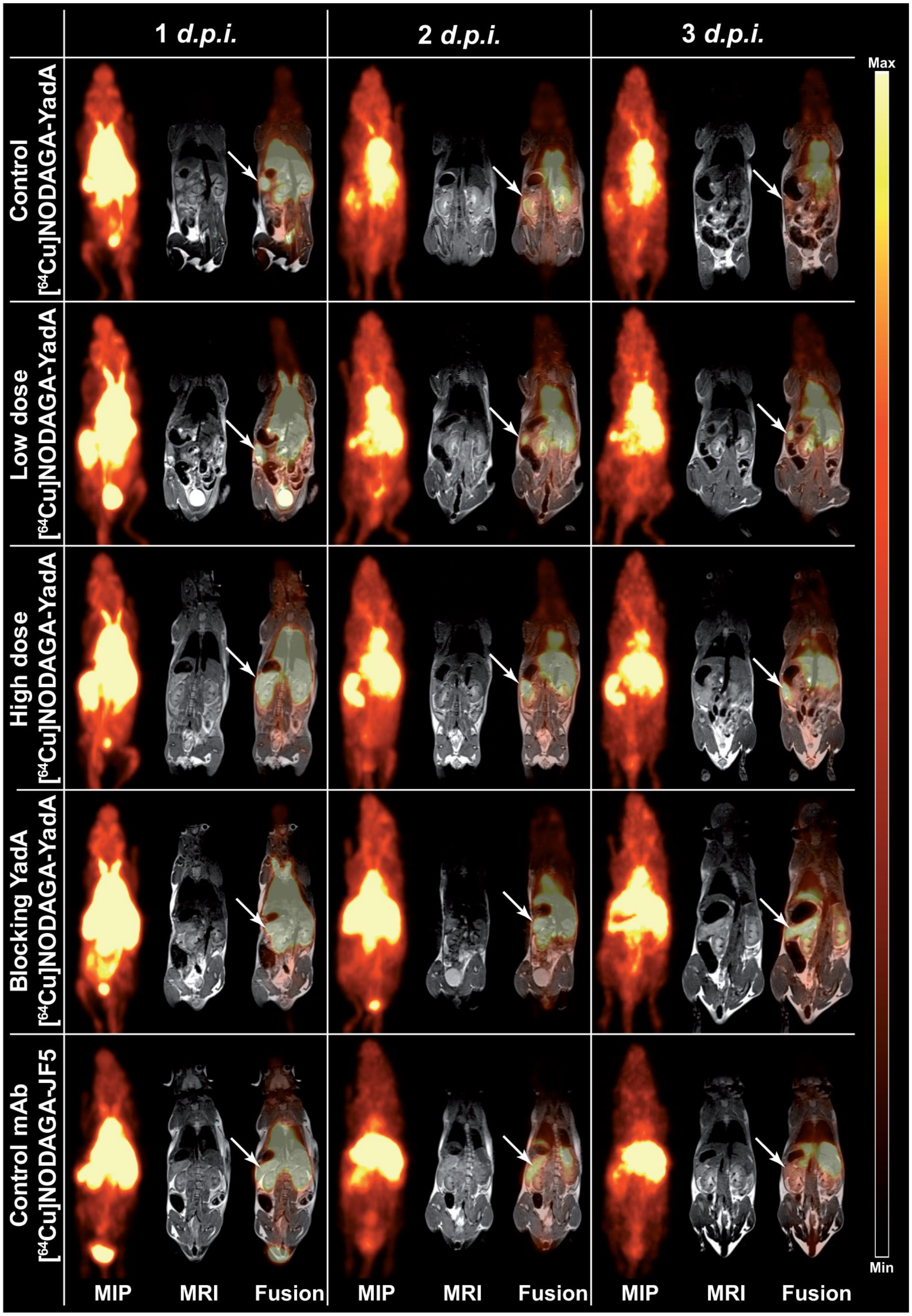

Supplemental Figure 3: Maximum intensity projections (MIP), coronal [^{64}Cu]NODAGA-YadA PET and fused PET and MR images (fusion) from PBS treated control, low and high dose infected mice one to three *d.p.i.* Administration of polyclonal non-radiolabeled antibody YadA 3 h prior the injection of the [^{64}Cu]NODAGA-YadA (blocking YadA) or administration of the *Aspergillus*-specific tracer [^{64}Cu]NODAGA-JF5 (Control mAb) into high dose infected mice serving as controls. Arrows indicate the position of the spleen of the mice.

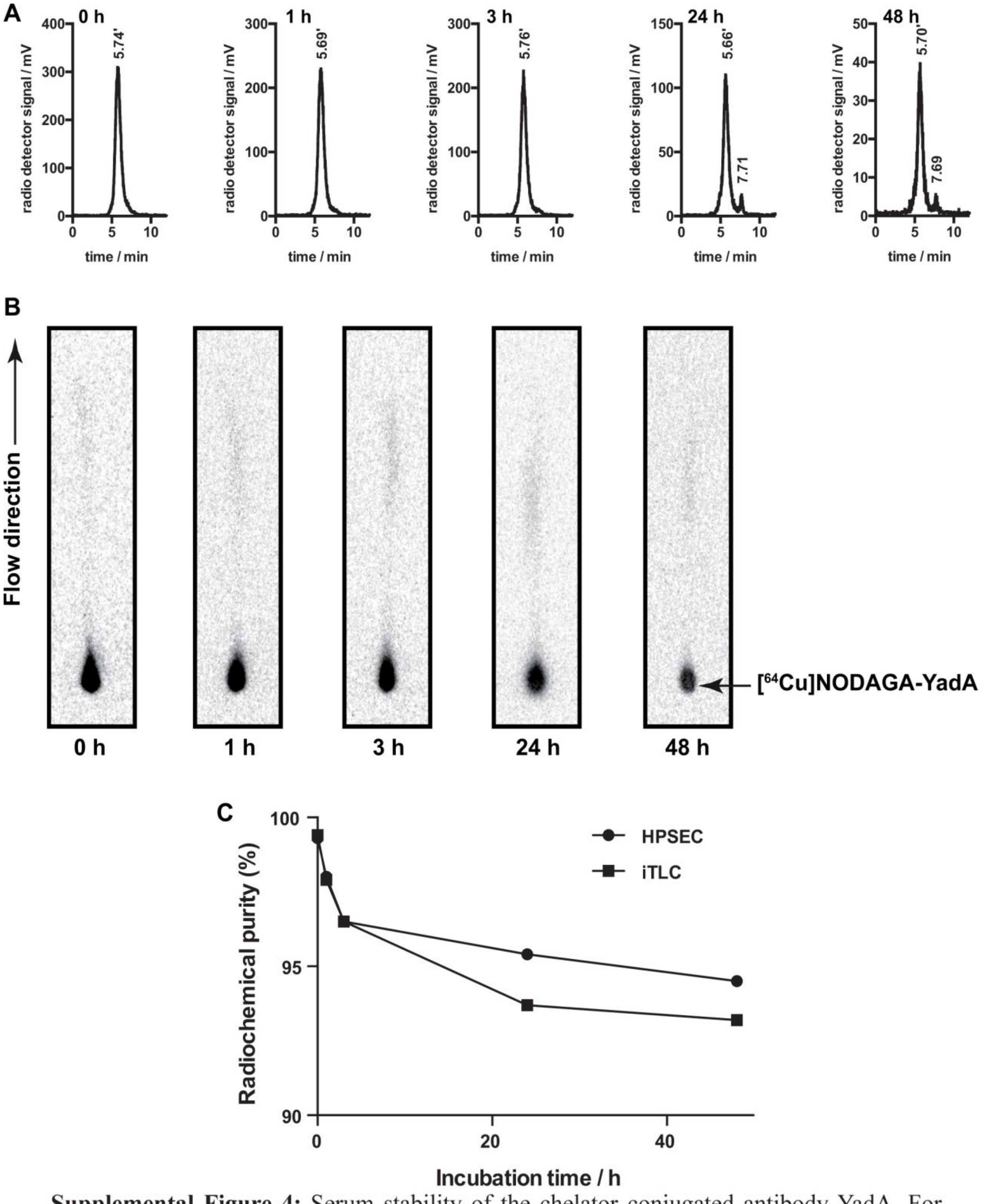

Supplemental Figure 4: Serum stability of the chelator conjugated antibody YadA. For assessment of the serum stability, one volume of [^{64}Cu]NODAGA-YadA (after clean-up with a Bio-Spin 6 column) was incubated with three volumes of C57BL/6 serum at 37 °C. Samples were removed after 0 h , 1 h, 3 h, 24 h and 48 h and immediately analyzed by radio-HPSEC (A). Retention times for void volume, reference IgG and internal volume were 3.34 min, 5.67 min and 8.82 min, respectively. In addition, samples were run on iTLC-SG paper with 0.1 M sodium citrate pH 5 and analyzed by autoradiography (B). The radiochemical purity over time with these two methods is shown in (C). The analysis shows no signs of proteolytic degradation, protein aggregation or copper transchelation to serum proteins over the time of 48 h under these conditions.

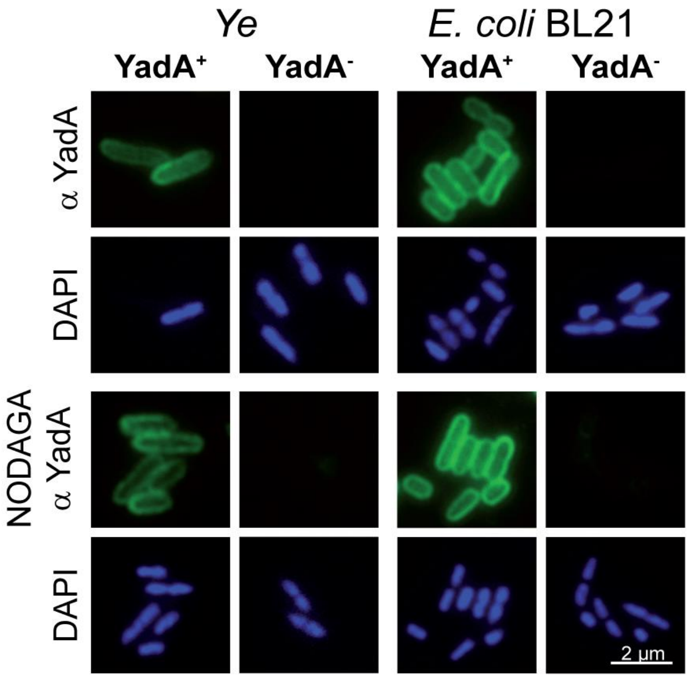

Supplemental Figure 5: Specificity of NODAGA-labeled YadA antibody

Immunofluorescence analysis of Ye wild type strain (YadA^{+}) or Ye YadA deficient mutant strain (YadA^{-}) or *E. coli* expressing or not expressing YadA. Bacteria were stained using either YadA antibody or NODAGA-YadA antibody followed by goat anti-rabbit secondary antibody and counterstained with DAPI. Representative pictures of 2 independent experiments are shown.

Supplementary Table 1: *In vivo* biodistribution of [^{18}F]FDG in PBS treated controls (group 1), low dose (group 2) and high dose *Y. enterocolitica* infected mice. Averages and standard deviations are given for various organs from ROIs of the PET images. Static 10 min PET scans were acquired 1, 2 and 3 *d.p.i.* Errors indicate one standard deviation. 10-12 MBq of [^{18}F]FDG was administered to each mouse.

[^{18}F]FDG, %ID/cc (± 1 SD)

Group	Acquisition	Spleen			Liver			Spine			Brain			Muscle		
	1	4.48	±	2.54	4.10	±	2.00	5.26	±	2.36	6.52	±	2.13	2.60	±	1.34
1	2	3.89	±	0.74	3.88	±	1.13	5.58	±	0.62	6.71	±	1.79	2.29	±	0.46
	3	3.16	±	0.41	3.14	±	0.86	4.54	±	0.35	5.97	±	0.71	2.11	±	0.82
	1	4.06	±	0.59	3.48	±	0.85	4.66	±	1.03	6.13	±	0.69	2.09	±	0.52
2	2	3.23	±	0.73	3.11	±	0.48	4.49	±	1.66	4.74	±	1.06	2.26	±	0.80
	3	3.61	±	0.80	2.92	±	0.78	3.76	±	0.92	5.78	±	1.82	2.29	±	0.68
	1	12.18	±	2.66	6.10	±	1.41	7.12	±	1.13	10.19	±	1.06	1.85	±	0.41
3	2	10.93	±	3.95	4.40	±	0.74	7.82	±	2.33	7.18	±	0.97	2.99	±	0.68
	3	9.89	±	3.83	4.06	±	1.03	8.34	±	1.75	6.41	±	1.42	3.04	±	0.66

Supplementary Table 2: *Ex vivo* biodistribution of organs of PBS treated control, low dose and high dose infected animals. Enhanced [^{18}F]FDG uptake in spleens of the high dose infected mice is seen 3 *d.p.i* with significant differences to the control and low dose infected animals.. Data are given as means ±1 SD or as spleen-to-muscle ratios.

[^{18}F]FDG Organ	PBS-treated mice (n = 3)			Low dose infected mice (n = 10)			High dose infected mice (n = 9)		
Blood	1.2	±	0.2	1.0	±	0.3	0.6	±	0.2
Spleen	7.7	±	1.1	6.7	±	2.1	22.8	±	6.1
Liver	2.3	±	0.5	2.8	±	1.0	4.1	±	1.2
Muscle	1.5	±	0.2	1.7	±	0.7	1.3	±	0.5
Spleen/muscle	5.2			4.0			18.0		

Supplementary Table 3: *In vivo* biodistribution of the *Y. enterocolitica* specific [^{64}Cu]NODAGA-YadA polyclonal antibody in PBS treated control, low dose and high dose *Y. enterocolitica* infected mice. Administration of polyclonal non-radiolabeled antibody YadA 3 h prior the injection of the [^{64}Cu]NODAGA-YadA (blocking YadA) or administration of the *A. fumigatus* specific tracer [^{64}Cu]NODAGA-JF5 (Control mAb) into high dose infected mice serving as controls. Averages and standard deviations are given for various organs from ROIs of the PET images. Static 10 min PET scans were acquired 1, 2 and 3 *d.p.i*. Errors indicate one standard deviation. 12-14 MBq of the respective antibody was administered to each mouse.

[^{64}Cu]NODAGA-YadA, %ID/cc (± 1 SD)

Group	Acquisition	Spleen	Liver	Spine	Brain	Muscle
	1	7.56 ± 0.98	9.95 ± 1.68	3.17 ± 0.62	1.29 ± 0.21	2.07 ± 0.45
1	2	6.83 ± 1.46	8.11 ± 0.39	2.75 ± 0.41	0.96 ± 0.09	2.34 ± 0.14
	3	6.84 ± 0.99	7.18 ± 0.86	2.83 ± 0.11	0.96 ± 0.12	2.16 ± 0.20
	1	11.30 ± 1.68	10.19 ± 1.30	3.69 ± 0.44	1.20 ± 0.18	2.25 ± 0.75
2	2	11.15 ± 1.19	9.03 ± 1.18	2.92 ± 0.83	1.07 ± 0.20	2.52 ± 0.33
	3	11.44 ± 0.84	8.02 ± 0.76	3.39 ± 0.46	0.99 ± 0.28	2.33 ± 0.32
	1	12.60 ± 1.37	9.38 ± 0.47	4.12 ± 0.51	1.15 ± 0.03	2.29 ± 0.32
3	2	12.87 ± 0.85	8.04 ± 0.46	3.93 ± 0.67	0.98 ± 0.10	2.95 ± 0.24
	3	11.00 ± 1.19	7.63 ± 0.54	3.45 ± 0.37	0.81 ± 0.08	2.83 ± 0.28
	1	10.50 ± 0.94	14.61 ± 0.91	3.86 ± 0.30	1.30 ± 0.18	2.02 ± 0.75
4	2	9.59 ± 2.03	11.08 ± 1.41	3.21 ± 0.88	1.00 ± 0.15	2.07 ± 0.33
	3	8.83 ± 1.33	8.95 ± 0.60	2.94 ± 0.23	0.83 ± 0.23	2.20 ± 0.32
	1	7.39 ± 0.67	13.81 ± 0.67	3.19 ± 0.81	0.84 ± 0.09	1.28 ± 0.32
5	2	5.25 ± 0.31	10.67 ± 1.11	2.47 ± 0.15	0.54 ± 0.05	1.47 ± 0.24
	3	4.30 ± 0.39	9.78 ± 0.45	2.19 ± 0.28	0.60 ± 0.05	1.29 ± 0.28

Supplementary Table 4: *Ex vivo* biodistribution of the *Y. enterocolitica* specific [^{64}Cu]NODAGA-YadA polyclonal antibody in PBS treated controls, low dose and high dose *Y. enterocolitica* infected mice. Averages and standard deviations are given for various organs. Spleens of low dose and high dose infected animals had significantly higher uptake of the tracer compared to the control and blocking groups and confirmed the PET findings. Errors indicate one standard deviation. 10-12 MBq of the respective antibody was administered to each mouse.

	[^{64}Cu]NODAGA-YadA, %ID/g (± 1 SD)														
Organ	Control			Low dose			High dose			Blocking			Control mAb JF5		
Blood	19.9	±	1.6	17.9	±	2.4	13.3	±	1.2	17.0	±	5.5	9.1	±	0.3
Spleen	13.2	±	1.7	29.5	±	3.9	24.8	±	3.0	17.1	±	2.8	13.9	±	1.8
Spine	2.6	±	0.3	3.6	±	0.5	4.2	±	0.3	3.7	±	0.3	2.5	±	0.1
Liver	7.1	±	0.9	10.0	±	1.1	10.9	±	0.9	14.0	±	1.2	18.7	±	1.4
Heart	5.6	±	0.7	5.3	±	0.7	4.9	±	0.7	6.4	±	1.4	4.3	±	0.4
Kidney	9.9	±	0.7	8.4	±	4.7	9.1	±	0.9	14.7	±	3.0	11.2	±	0.8
Stomach	2.9	±	0.6	3.4	±	0.3	4.0	±	0.8	4.1	±	0.4	2.9	±	0.3
Colon	3.0	±	0.6	3.9	±	0.4	4.3	±	0.3	4.9	±	0.6	2.9	±	0.3
Muscle	1.8	±	0.2	2.0	±	0.3	2.9	±	0.3	2.3	±	0.5	1.3	±	0.1
Brain	0.5	±	0.1	0.5	±	0.1	0.5	±	0.0	0.6	±	0.1	0.5	±	0.0
